# A comparison of the Thunderbeat and standard electrocautery devices in head and neck surgery: a prospective randomized controlled trial

**DOI:** 10.1007/s00405-021-06739-z

**Published:** 2021-03-19

**Authors:** N. C. Kuipers, B. J. de Kleijn, J. Wedman, B. F. A. M. van der Laan, B. E. C. Plaat, G. B. Halmos

**Affiliations:** 1 Department of Otorhinolaryngology, Head and Neck Surgery, University of Groningen, University Medical Center Groningen, Groningen, The Netherlands; 2 Utrecht University, Utrecht, The Netherlands; 3 Department of Otorhinolaryngology, Head and Neck Surgery, Radboud University Medical Center, Nijmegen, The Netherlands; 4 Department of Otorhinolaryngology, Head and Neck Surgery, Haaglanden Medical Center, The Hague, The Netherlands

**Keywords:** Adverse event, Energy based device, Neck dissection, Operative time, Thunderbeat, Total laryngectomy

## Abstract

**Purpose:**

New energy-based sutureless vessel ligation devices, such as the Thunderbeat (Olympus Medical Systems Corp., Tokyo, Japan), could reduce operative time and limit blood loss in head and neck surgery; however, efficacy and safety in major head and neck surgery have not been investigated in a prospective, randomized study.

**Methods:**

This prospective, double-arm, randomized controlled trial consisted of two parts: total laryngectomy (TL) and neck dissection (ND). Thirty patients planned for TL were randomized in two groups. For the ND part, forty-two operative sides were likewise randomized. In both parts, Thunderbeat was used in addition to the standard instrumentation in the intervention groups, while only standard instrumentation was used in the control groups. Primary outcome values were blood loss, operative time and complication rate.

**Results:**

For the TL part there was no difference in mean blood loss (*p* = 0.062), operative time (*p* = 0.512) and complications (*p* = 0.662) between both hemostatic techniques. For the neck dissection part, there was a reduction in blood loss (mean 210 mL versus 431 mL, *p* = 0.046) and in operative time (median 101 (IQR 85–130) minutes versus 150 (IQR 130–199) minutes, *p* = 0.014) when Thunderbeat was used. There was no difference in complication rate between both hemostatic systems (*p* = 0.261).

**Conclusion:**

The Thunderbeat hemostatic device significantly reduces operative blood loss and operative time for neck dissections, without increase in complications. In TL, blood loss using Thunderbeat was comparable with the standard technique, but the operative time tended to be shorter.

**Trial registration:**

UMCG Research Register, Reg. no. 201700041, date of registration: 18/1/2017

## Introduction

A total laryngectomy (TL) with additional neck dissection (ND) is considered as major head and neck surgery. During head and neck surgery, precise hemostasis ensures a clear view on the surgical field. Minimalizing blood loss and operative time are beneficial both for the patient and cost-effectiveness [1]. Traditional instruments for dissecting and controlling hemostasis during head and neck surgery are bipolar forceps and monopolar scalpel, with the additional ligation or clipping for larger vessels. Some operative time is lost due to switching between operative instruments. Reduced operative time can be expected if the same device is used for both dissection and hemostasis.

The rate of overall complications during TL is estimated at 45% [2]. Because of the development of organ preserving strategies, an increased number of patients undergo a salvage TL after initial (chemo)radiation instead of primary TL. This is associated with a higher risk in complications of 42–67.5% [3, 4]. Post-operative complications entail bleeding, dysphagia, stomal complications and airway issues. The most common, serious and difficult to treat complication of TL is pharyngocutaneous fistula. Its incidence has a wide range from 3–65% [5]. It has a grave effect on postoperative functional recovery and quality of life. It usually leads to increased hospitalization and can lead to re-operations, decreased speech and swallowing function.

Complication rates for ND vary greatly according to the extent of the surgery and the levels involved [6], especially in salvage surgery [7]. Beside the general surgical complications, like bleeding and postoperative wound infection, ND can lead to loss of range of motion and muscle impairments of the shoulder due to sacrificing or damaging accessory nerve, loss of sensation, lymphedema and pain [4, 6, 8]. More effective hemostasis and a consequently better view on the surgical field may lead to less complications.

To achieve effective hemostasis during operation, new energy-based devices (EBD) have been developed, e.g., the advanced bipolar LigaSure Small Jawclamp (Medtronic) and the ultrasonic Harmonic Focus scalpel (Ethicon Endo-Surgery, Cincinnati, OH, USA). These devices are multi-purpose. Dissection, hemostasis and cutting can be performed with the same instrument, saving operative time. THUNDERBEAT Open Fine Jaw (TB, Olympus Medical Systems Corp., Tokyo, Japan), however, has a hybrid blade that combines both ultrasonically cutting, with bipolar coagulation. This combination enables coagulation and cutting at the same time [9–11]. It can be used for sealing and ligation of vessels up to a diameter of 7 mm.

EBD have been clinically evaluated for minimal invasive gynecological surgery, open thyroidectomy and free flap reconstructive surgery for the head and neck area. These studies suggest a decrease in hospital stay, blood loss and postoperative drainage [12–14]. However, there are no clinical studies evaluating TL and ND. We have, therefore, conducted a randomized controlled study to evaluate the safety and efficacy of the TB during total laryngectomy surgery and neck dissection in comparison with standard electrocautery devices (SED).

## Methods

### Ethical considerations

The study was checked by the Institutional Review Board of the University Medical Center Groningen (UMCG) and judged as according to the Dutch Medical Research Law, there is no need for Institutional Review Board approval; therefore, a waiver was released. The study was performed in accordance with Good Clinical Practice principles and the Consolidated Standards of Reporting Trials (CONSORT) statement.

### Patient selection

For the TL part, consecutive patients with primary or recurrent laryngeal or hypopharyngeal cancer, scheduled for TL between Feb 2018 and Jun 2019 at a single tertiary center (UMCG, the Netherlands), were eligible for inclusion. During the same period at the same center, consecutive patients with head or neck cancer, scheduled for Radical Neck Dissection (RND), Modified Radical Neck Dissection (MRND) or Selective Neck Dissection (SND) were eligible for inclusion in the ND part. Selective neck was performed only in 2 patients in both groups and the surgery involved level 2–5 in all four patients (only level 1 was spared).

### Randomization and blinding

For both parts of the study, computer-generated randomization was used to allocate patients equally in two groups. After allocation, the intervention group was operated on with the additional use of TB, while the control group was operated on with use of SED only.

Patients who underwent a ND in combination with TL were enrolled in both parts. Surgical staff and data assessors were not blinded.

### Pre-operative data

The following characteristics were acquired and analyzed: gender, age, comorbidity based on the Adult Comorbidity Evaluation 27 (ACE-27) score [15], use of anti-coagulants pre-operatively and operatively (except for the use of prophylactic low molecular weight heparin), tumor site, previous treatment on surgical site, salvage surgery, pTNM-stage and disease stage.

### Surgical technique

Patients were treated in accordance with the treatment protocol of the medical center. All procedures were performed by four senior head and neck oncologic surgeons.

Standard devices for cutting and hemostasis included monopolar and bipolar energy devices (ERBE GmbH, Germany), sutures and surgical clips. In the TB arm, the TB device was set at SEAL/CUT ‘1’ and SEAL ‘3’. Intra-operative blood loss was measured by weighing the gauzes per-operatively and subtracting the weight of the gauzes. One gram was assumed equal to one mL blood. Operative time of the whole procedure was recorded, as well as operative time of (hemi) thyroidectomy during TL.

### Outcomes measures

Primary outcome measures were operative time, blood loss and post-operative complications (according to the Clavien–Dindo (CD) classification [16]). Major bleeding was defined as a bleeding that requires surgical intervention. The CD system is a validated classification system, grading postoperative surgical complications from grade I (minor deviation from normal postoperative course), through grade III (complications requiring a surgical intervention) to grade V (death of a patient). Secondary outcome measures were time interval between surgery and drain removal, post-operative drainage volume and length of hospital stay.

### Statistical methods

Data was clustered for analysis if possible. For both parts, comorbidity score was clustered as ‘None/Mild’ (ACE-27 0–1) and ‘Moderate/Severe’ (ACE-27 2–4); pathological tumor stage was clustered as ‘Early disease’ (T1–T2) and ‘Advanced disease’ (T3–T4). For the TNM classification, the 8th edition of the UICC was used [17]. In all cases the pathological TNM (pTNM) was used; if it was not available, the clinical TNM (cTNM) was used. Results of complications (based on CD-classification) was clustered into no complications (0–1) and mild/major (2–5) complications.

For the TL part the site of primary tumor was clustered as larynx or pharynx; salvage surgery included cases with recurrent/residual disease after (chemo) radiation and also surgery for a dysfunctional larynx without signs of malignancy. For the ND part no clusters were made.

The distribution of continuous variables was assessed by the Shapiro–Wilk test. For normal distributed variables a mean and a standard deviation (SD) were assessed, for variables without normal distribution, the median and inter quartile range (IQR). Significance was determined for all continuous variables using the Mann–Whitney *U* test. For dichotomous nominal or categorical variables, a Chi-squared test was used to assess significance. In tests with a count > 20% below 5, the Fisher’s exact test was used. A *p* value of less than 0.05 was considered significant. All analysis were performed using IBM SPSS Statistics for Mac OS, 64-bit edition [18].

## Results

### TL part results

**Fig. 1:**
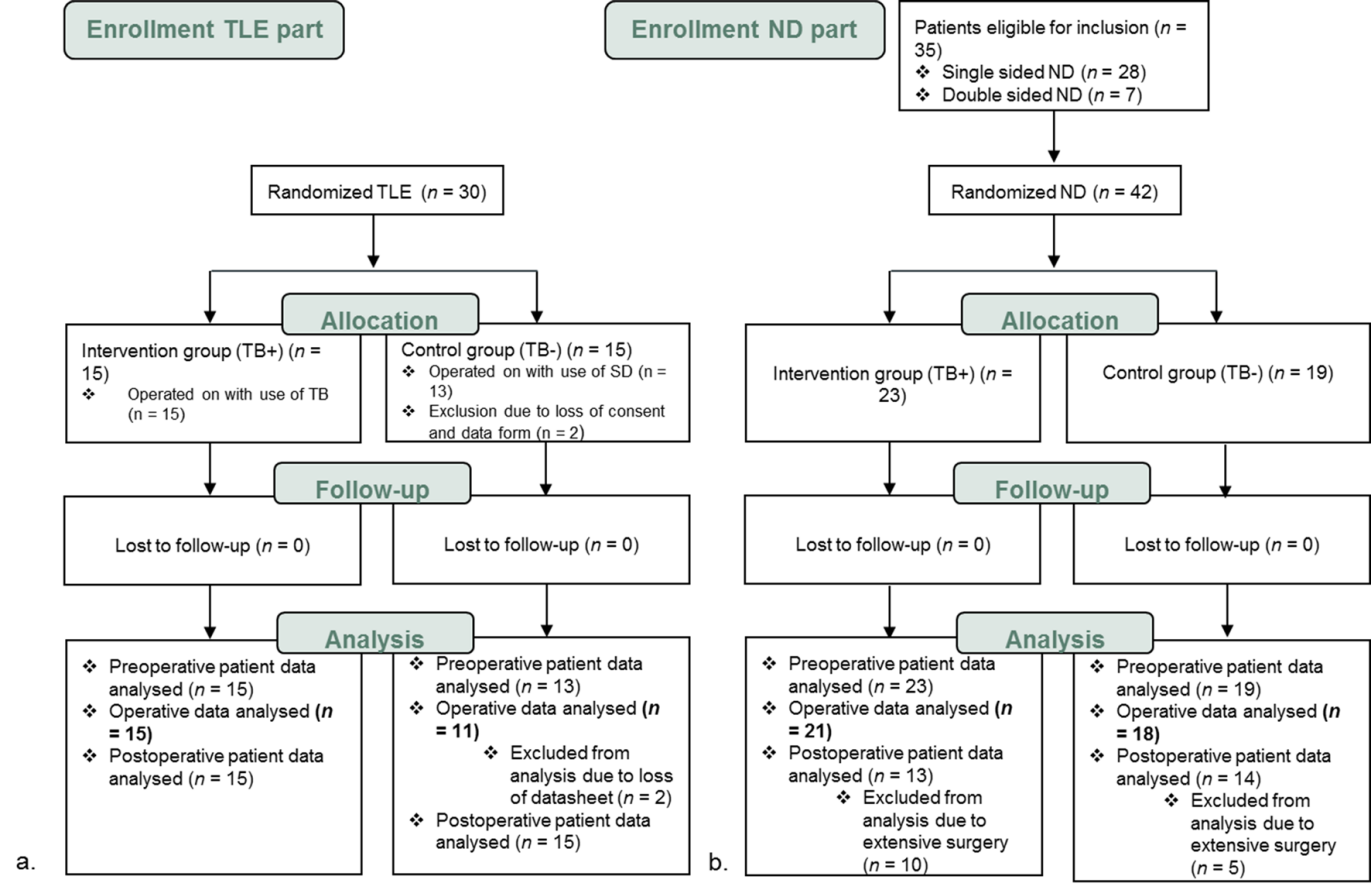
Flowchart diagram of included patients in both parts of the study

Figure 1a shows the process of patient enrolment and analysis. This part prospectively randomized thirty patients with the indication for total laryngectomy, with or without additional neck dissection and reconstructive surgery, in two groups. As shown in Fig. 1a, the intervention group consisted of fifteen patients and eleven patients in the control group. The demographic characteristics of the patients, tumor site and stage for both groups are listed in Table 1. The two groups were comparable for gender, age, comorbidity score, pre- and perioperative use of anticoagulants, tumor site, salvage surgery, T stage, N stage and disease stage. Significance for ‘previous treatment’ was not calculated due to small sample size.

**Table 1:** Overview and comparison of preoperative clinical characteristics of the two TL groups

Patient	TB group,* n* = 15	SED group,* n* = 13	*p* value
Gender *n *(% of total)			
Female	5 (33.3%)	5 (38.5%)	1.000^e^
Male	10 (66.6%)	8 (61.5%)	
Age mean (range)			
Years	64.00 (39–86)	65.77 (47–81)	0.618^f^
Comorbidity (based on ACE-27 score)* n* (% of total)			
None/mild (0–1)	9 (60%)	5 (38.5%)	0.256^g^
Moderate/severe (2–4)	6 (40%)	8 (61.5%)	
Preoperative anti-coagulation use *n *(% of total)			
Yes	1 (6.7%)	4 (30.8%)	0.153^e^
No	14 (93.3%)	9 (69.2%)	
Anti-coagulation during surgery *n *(% of total)			
Yes	1 (6.7%)	3 (23.1%)	0.311^e^
No	14 (93.3%)	10 (76.9%)	
Oncology			
Site *n* (% of total)			
Larynx	10 (66.6%)	8 (61.5%)	1.000^e^
Pharynx	5 (33.3%)	5 (38.5%)	
Previous treatment *n *(% of total)			
No or TOLS	9 (60%)	4 (30.8%)	^d^
RT	3 (20%)	6 (46.2%)	
ChRT	2 (13.3%)	2 (15.4%)	
RT for other head and neck primary	1 (6.7%)	1 (7.7%)	
Salvage surgery^a^ *n *(% of total)			
No	8 (53.3%)	4 (30.8%)	0.229^g^
Yes^b^	7 (46.7%)	9 (69.2%)	
pTNM8^c^* n *(% of total)			
T1–2 (early)	0 (0%)	2 (50%)	0.091^e^
T3–4 (advanced)	8 (100%)	2 (50%)	
N0	4 (50%)	2 (50%)	1.000^e^
N +	4 (50%)	2 (50%)	
Stage *n *(% of total)			
Early	0 (0%)	2 (50%)	0.110^e^
Advanced	8 (100%)	2 (50%)	

^a^Including primary TL after previous RT for other tumor

^b^Including salvage TL for afunctional larynx after RT: 1 patient in TB + , 3 patients in TB− (*p* = 0.585)

^c^Excluding salvage surgery and afunctional larynx

^d^Significance not determined due small sample size

^e^Fisher’s exact test

^f^Mann–Whitney *U* test

^g^Chi-squared test

#### Operative results

As shown in Table 2, no statistically significant differences of the primary and secondary outcome measures were found between the two cohorts. However, intraoperative blood loss was about half for the TB group compared to the SED group, for both the complete TL procedure (*p* = 0.06) and the (hemi) thyroidectomy procedure (*p* = 0.755). After excluding an outlier in the SED group, this result is not significantly different (TB + : 214 ± 203 mL, TB-: 368 ± 324 mL, *p* = 0.123 ±). In patients undergoing TL, with use of the TB, operative time had a slightly higher median and IQR compared to the SED group. For the thyroid surgery, a lower, but insignificant median and IQR for operative time was observed for the TB group.

**Table 2:** Overview and comparison of outcomes between the two TL groups

Intraoperative outcomes		TB group,* n* = 15	SED group,* n* = 11	*p *value
Operative time median (IQR)				
Total laryngectomy (minutes)		108 (72–139)	91 (64–132)	0.512^c^
Thyroid surgery (minutes)		13 (11–17)	20 (9–44)	0.731^c^
Blood loss mean ± SD				
During TL (mL)		214 ± 203	572 ± 713	0.062^c^
During thyroid surgery (mL)		19 ± 30	39 ± 62	0.755^c^
Major bleed *n *(% of total)				
Yes		0 (0%)	1 (7.7%)	0.464^b^
No		15 (100%)	12 (9.3%)	
Follow-up outcomes^a^				
CD-score *n *(% of total)				
0–1		7 (46.7%)	5 (38.5%)	0.662^b^
4–5		8 (53.3%)	8 (61.5%)	
Hospital stay mean ± SD				
Days		20 ± 24	18 ± 15	0.274^c^
Time until first drain removal mean ± SD				
Days		3.0 ± 1.0	2.9 ± 1.0	0.683^c^
Post-operative drainage volume median (IQR)				
Milliliters		205 (104–468)	160 (106–220)	0.254^c^

Distribution of data was normal for: ‘Blood loss’, ‘Hospital stay’ and ‘Time until first drain removal’

Major bleeding was defined as a bleeding that requires surgical intervention

*IQR* inter quartile range, *SD* standard deviation, *CD-score* Clavien–Dindo classification score

^a^*n* = 13 for SED group

^b^Fisher’s exact test

^c^Mann–Whitney *U* test

#### Postoperative results

As shown in Table 2 the complication rate in the TB group was comparable with the SED group (53.3% compared to 61.5%). Hospital stay and time interval between surgery and first drain removal did not differ significantly. The post-operative drainage volume was insignificantly higher in the TB group; however, it did not result in statistically significant longer time until drain removal or extended hospitalization.

### ND part results

This part prospectively randomized forty-two ND procedures of thirty-five patients, with or without additional TL, parotidectomy or reconstructive surgery, in two groups (Fig. 1b). The intervention group consisted of twenty-three and the control group of nineteen NDs. Due to incomplete intra-operative datasheets for three patients, the surgical data was analyzed for twenty-one patients in the intervention group and eighteen in the control group.

The demographic characteristics of the patients, tumor characteristics and operative procedure details for both groups are listed in Table 3. The two groups were comparable for gender, age, comorbidity score, anti-coagulation use, disease stage and primary versus salvage surgery. Statistical significance for ‘histology’, ‘site of primary tumor’, ‘previous treatment’, ‘nodal stage’ and ‘type of ND’ was not calculated due to low sample size.

**Table 3:** Overview and comparison of preoperative clinical characteristics of the two ND groups

Patient	Thunderbeat group,* n* = 23	Standard devices group, *n* = 19	*p* value
Gender *n *(% of total)			
Female	9 (39.1%)	4 (21.1%)	0.207^d^
Male	14 (60.9%)	15 (78.9%)	
Age mean (range)			
Years	62.78 (39–79)	61.68 (41–88)	0.518^c^
Comorbidity (based on ACE-27 score)* n *(% of total)			
None/Mild (0–1)	11 (47.8%)	9 (47.4%)	0.976^d^
Moderate/Severe (2–4)	12 (52.2%)	10 (52.6%)	
Anti-coagulation *n *(% of total)			
Yes	15 (65.2%)	15 (78.9%)	0.327^d^
No	8 (34.8%)	4 (21.1%)	
Oncology			
Histology *n *(% of total)			
SCC	21 (91.3%)	16 (84.2%)	*
Melanoma	1 (4.3%)	2 (10.5%)	
Adenocarcinoma	1 (4.3%)	1 (5.3%)	
Site of primary tumour *n *(% of total)			
Unknown	2 (8.7%)	4 (21.1%)	*
Larynx and hypopharynx	7 (30.4%)	6 (31.6%)	
Oral	6 (26.1%)	5 (26.3%)	
Skin	7 (30.4%)	4 (21.1%)	
Glandula parotis	1 (4.3%)	0 (0%)	
Previous treatment *n *(% of total)			
No	19 (82.6%)	12 (63.2%)	*
RT^a^	3 (13.0%)	2 (10.5%)	
ChRT^a^	0 (0%)	1 (5.3%)	
Immunotherapy	0 (0%)	1 (5.3%)	
Previous surgery	1 (4.3%)	3 (15.8%)	
pTNM8 *n *(% of total)			
T1 – 2 (Early)	5 (38.5%)	4 (50%)	0.673^d^
T3 – 4 (Advanced)	8 (61.5%)	4 (50%)	
Tx, T0 excluded: TB + n = 8, TB− n = 13			
N0	3 (13.0%)	1 (5.3%)	*
N1	2 (8.7%)	1 (5.3%)	
N2	8 (34.8%)	7 (36.8%)	
N3	7 (30.4%)	8 (42.1%)	
Salvage** and Nx	3 (13.0%)	2 (10.5%)	
Operative procedure			
Type of ND *n* (% of total)			
MRND	21 (91.3%)	15 (78.9%)	*
RND	0 (0%)	2 (10.5%)	
SND	2 (8.7%)	2 (10.5%)	
TL and/or pectoral flap reconstruction *n* (% of total)			
Yes	10 (43.5%)	5 (26.3%)	0.248^d^
No	13 (56.5%)	14 (73.7%)	
Primary versus salvage surgery *n* (% of total)			
Salvage	3 (13.0%)	2 (10.5%)	0.802^d^
Primary	20 (87.0%)	17 (89.5%)	

*p value could not be calculated due to low count

^a^Included radiation of neck

^b^Fisher’s exact test

^c^Mann–Whitney U test

^d^Chi-squared test

#### Operative results

Regarding the primary outcomes, a significant difference was found between the two cohorts for operative time and blood loss during ND. Intra-operative blood loss was more than halved for the intervention group compared to the control group (TB + : 210 ± 209 mL, TB−: 431 ± 409 mL, *p* = 0.046). However, after exclusion of an outlier in the SED group, this result is not statistically significant (TB + : 210 ± 209 mL, TB−: 357 ± 269 mL, *p* = 0.078 ±). In patients undergoing ND with the use of TB, the median for operative time was forty-five minutes less than the control group [TB + : 101 (85–130) minutes, TB−: 150 (130–199), *p* = 0.014]. There were slightly more complications within the control group than the intervention group; however, this was not a statistically significant difference.

#### Postoperative results

Hospital stay, time interval between surgery and first drain removal and drainage volume did not differ statistically between the thirteen patients in the intervention group and fourteen patients in the control group (Table 4).

**Table 4:** Overview and comparison of outcomes between the two ND groups

Surgery	Thunderbeat group,* n* = 23	Standard devices group, *n* = 19	*p* value
Operative time^a^ median (IQR)			
Minutes	101 (85–130)	150 (130–199)	0.014^c^
Blood loss ^a^mean ± SD			
Millilitres	210 ± 209	431 ± 409	0.046^c^
Major bleed *n *(% of total)			
Yes	0 (0%)	1 (5.3%)	0.452^e^
No	23 (100%)	18 (94.7%)	

Follow-up	Thunderbeat group,* n* = 13	Standard devices group, *n* = 14	*p* value
Comorbidities^b^* n *(% of total)			
CD-score: 0–1	16 (69.6%)	10 (52.6%)	0.261^d^
CD-score: 4–5	7 (30.4%)	9 (47.3%)	
Hospital stay mean ± SD			
Days	13 ± 11	10 ± 7	0.304^c^
Time until first drain removal mean ± SD			
Days	4.4 ± 2.8	4.4 ± 1.7	0.553^c^
Total drainage fluid volume mean ± SD			
Millilitres	395 ± 264	466 ± 261	0.245^c^

Distribution of data was normal for: ‘Blood loss’, ‘Hospital stay’, ‘Time until first drain removal’ and ‘Total drainage fluid volume’

Major bleeding was defined as a bleeding that requires surgical intervention

*IQR* inter quartile range, *SD* standard deviation, *CD-score* Clavien Dindo classification score

^a^TB+: *n *= 21 TB−: *n *= 18

^b^TB+: *n *= 23 TB−: *n *= 19

^c^Mann-Whitney U test

^d^Chi-squared test

^e^Fisher’s exact test

## Discussion

This is the first study to analyze the effect of the TB device during major head and neck surgery, compared to SED in a prospective, randomized double-arm trial.

This study showed statistically significant reduction in operative time and blood loss when TB was used during ND. During TL, blood loss was reduced, but operative time was increased, although both not significantly when TB was used. As there was no difference in post-operative complications for both procedures, the TB can be regarded as a safe instrument for both TL and ND.

Perioperative blood loss and post-operative complications may lead to prolonged hospitalization, re-operation, delay in adjuvant therapy, etc. [1]. Studies by both Gambardella et al. concerning hemostasis in axillary lymph node dissection and Suzuki et al. concerning short-term outcomes for neck dissection, show a trend toward limiting blood loss with use of the TB and Ligasure in comparison with electrocautery [9, 10]. In line with these studies, we experienced a decrease in blood loss, however, statistically not significant. The differences within the TL part were likewise striking in term of blood loss and very likely statistically insignificant due to the small sample size.

Literature shows shorter operative time when using the TB compared to conventional electrosurgery in laparoscopic procedures, such as hysterectomy and lymphadenectomy [9, 19]. The contradictory results for the present TL part for operative time could be explained by several factors: limited sample size and the fact that the TB is less suitable in the most crucial part of the surgery, namely during the mucosal incisions. To ensure optimal healing of the mucosal wound, ultrasonic or bipolar energy is avoided when the pharyngeal mucosa in incised. However, during a specific part of the surgery, namely during dissection of the thyroid gland from the larynx, a beneficial effect of TB could be observed. For the ND, however, a clear benefit is shown in favor of TB. The difference in median operative time was almost fifty minutes, which could benefit the patient due to diminished anesthetics and increase cost-effectiveness. However, performing a reliable cost-effectiveness study was not possible, due to several factors. The costs of the operating room time highly differs among hospitals and the costs of the TB depends on an agreement with the distributor. However, despite of not giving exact cost calculations, the data of the present study may be used for cost calculations. Each professional can calculate the cost-effectiveness of purchasing such a device, by calculating the OR time costs and asking an offer from the local distributor.

Several studies have concluded that the TB can be as safe as other energy-based devices when used in laparoscopic surgery [20–22], thyroid surgery [11] and neck dissections [10]. This study has indicated that the TB is at least as safe as the SED during TL and ND, as the statistically insignificant difference was found in favor of the TB group.

The outcomes of this study regarding hospitalization seem comparable to earlier study results. In other studies, analyzing new energy-based devices within a range of surgical procedures, the duration of hospitalization was either the same or less. In a prospective trial regarding the efficacy of the UltraCision Harmonic scalpel in thyroidectomy by Papavramidis et al. a statistically significant shortening of hospitalization was observed of almost a day [13]. However, a meta-analysis of three RCTs also analyzing hospitalization after neck dissection with use of the Harmonic scalpel versus SED, concluded that the marginal shortening they found was not statistically significant [23]. In a retrospective non-inferiority study by van Slycke et al. investigating the hospital stay for patients undergoing a TB-assisted thyroidectomy versus SED, stated that the TB was non-inferior to SED for the aspect of hospitalization [11]. Fagotti et al. prospectively compared patients undergoing laparoscopic hysterectomy TB-assisted with SED, with no difference (*p* = 0.82) in duration hospitalization [19]. In this study, there was no statistically significant differences in hospital stay for the TB group in both arms. Interestingly, the remarkably lower blood loss had no measurable impact on hospital stay or complication, which is also very likely due to the limited sample size. To establish a certain non-inferiority or superiority, a larger sample size is required. However, the presented data prove that TB is safe (no more bleeding than operating with SED). On the other hand, it also gives honest results to prevent too high expectations of using TB.

### Strengths and limitations

The strengths of the present study lie in its prospective, randomized design, evaluating two of the most common and elaborate procedures in head and neck surgery. During the study, treatment protocols were not changed. Furthermore, the same four skilled surgeons performed the surgery in all patients, limiting the learning curve for the use of the TB device to a minimum. Unfortunately, due to unforeseen circumstances, there was a loss of intra-operative data in several cases. The results have to be interpreted with caution, as this unblinded study included a limited number of patients and there is high deviation of the data (for instance for the blood loss).

## Conclusions

This study demonstrates the safety of Thunderbeat compared to standard electrocautery devices in TL and ND. TB significantly reduces operative time and operative blood loss during ND. This effect could not be seen in TL surgery; however, the blood loss was reduced with more than 50% during surgery with TB versus SED. Due to the limited sample size, the results need to be carefully interpreted and larger, randomized, powered studies are necessary to confirm the suggested trend for blood loss in TL and establish whether the TB could further diminish operative time in major head and neck surgery.

## Data Availability

The authors affirm that this manuscript is an honest, accurate, and transparent account of the study being reported; that no important aspects of the study have been omitted; and that any discrepancies from the study as planned have been explained. The data that support the findings of this study are available on request from the corresponding author.
